# HIV knowledge, self-perception of HIV risk, and sexual risk behaviors among male Tajik labor migrants who inject drugs in Moscow

**DOI:** 10.1186/s12889-023-17543-1

**Published:** 2024-01-11

**Authors:** Casey Morgan Luc, Judith Levy, Mahbat Bahromov, Jonbek Jonbekov, Mary E. Mackesy-Amiti

**Affiliations:** 1 University of Illinois Chicago, Chicago, USA; 2 PRISMA Research Center, Dushanbe, Tajikistan

**Keywords:** HIV Risk Self-Perception, HIV Knowledge, Psychosocial Factors, Sexual risk behavior, Injection drug use, Tajik migrant worker

## Abstract

**Background:**

The interplay of human immunodeficiency virus (HIV) knowledge and self-perception of risk for HIV among people who inject drugs is complex and understudied, especially among temporary migrant workers who inject drugs (MWID) while in a host country. In Russia, Tajik migrants make up the largest proportion of Moscow’s foreign labor. Yet, HIV knowledge and self-perceived risk in association with sexual risk behavior among male Tajik MWID in Moscow remains unknown.

**Objective:**

This research examines knowledge about HIV transmission, self-perception of HIV risk, and key psychosocial factors that possibly contribute to sexual risk behaviors among male Tajik labor MWID living in Moscow.

**Methods:**

Structured interviews were conducted with 420 male Tajik labor MWID. Modified Poisson regression models investigated possible associations between major risk factors and HIV sexual risk behavior.

**Results:**

Of the 420 MWID, 255 men (61%) reported sexual activity in the last 30 days. Level of HIV knowledge was not associated in either direction with condom use or risky sexual partnering, as measured by sex with multiple partners or female sex workers (FSW). Lower self-perceived HIV risk was associated with a greater likelihood of sex with multiple partners (aPR: 1.79, 95% CI: 1.34, 2.40) and FSW (aPR: 1.28, 95% CI: 1.04, 1.59), but was not associated with condom use. Police-enacted stigma was associated with sex with multiple partners (aPR: 1.22, 95% CI: 1.01, 1.49) and FSW (aPR: 1.32, 95% CI: 1.13, 1.54). While depression and lower levels of loneliness were associated with condomless sex (aPR: 1.14, 95% CI: 1.05, 1.24; aPR: 0.79, 95% CI: 0.68, 0.92, respectively), only depression was associated with condomless sex with FSW (aPR: 1.26, 95% CI: 1.03, 1.54).

**Conclusions:**

HIV prevention programing for male Tajik MWID must go beyond solely educating about factors associated with HIV transmission to include increased awareness of personal risk based on engaging in these behaviors. Additionally, psychological services to counter depression and police-enacted stigma are needed.

## Introduction

Research has shown that labor migration from low to high human immunodeficiency virus (HIV) prevalence countries is associated with increased risk of HIV infection for migrant workers [1, 2] This risk is further heightened among migrants who inject drugs (MWID) while in the host country [3]. In Russia, a country with high HIV prevalence [4], HIV risk for MWID is exacerbated by disparities in the availability and provision of HIV prevention and treatment services for labor migrants [3, 5–7]. In addition, MWID are impacted negatively by Russian policing practices that focus on enforcing petty criminal justice penalties for migrants in general and toward MWID in particular through physical harassment and syringe confiscation to discourage drug use. Such practices hinder harm reduction efforts and create barriers for MWID in accessing services for the prevention, treatment and care of HIV [8, 9].

This research presents findings on the prevalence and correlates of HIV sexual risk behavior among male Tajik MWID who are temporarily living and working in Moscow. While in Moscow, MWID experience the “double jeopardy” of social marginalization due to their dual status as a migrant and injection drug user, both of which are thought to increase sexual risk for HIV [3, 10]. Meanwhile, HIV knowledge and HIV risk perception have been found to influence sexual risk behavior among those who inject drugs [11, 12], but the interplay of these two factors on sexual risk behavior is complex [13], and less understood among people who inject drugs (PWID). One cross-sectional study conducted with PWID in Iran found that HIV risk perception modified the association between HIV knowledge and sexual risk behavior, but the study sample was small and not generalizable [14]. A separate cross-sectional study partly comprised of PWID in Iran identified a “relatively good” level of comprehensive knowledge of HIV and acquired immunodeficiency syndrome (AIDS), but the prevalence of HIV risk perception was low while sexual risk behavior was high [15]. These findings suggest that engaging in risky sexual behavior may be best explained by self-perception of being at HIV risk rather than degree of HIV knowledge alone. Whether through effect modification or not, the relationship between HIV risk perception and HIV knowledge on sexual risk behavior has yet to be investigated and reported in the scientific literature among MWID.

In addition, psychosocial factors appear to influence sexual risk behavior among PWID while possibly playing a role in promoting sexual risk behavior among MWID [11, 12, 15]. Stigma has been well-documented to undermine progress towards ending the global HIV/AIDS epidemic, but the complexity of intersectional stigma calls for research to better measure and contextualize stigma in separate global populations [16]. Due to the experience of aggressive policing tactics and harassment, migrants are particularly prone to police-enacted stigma that may form a “driving force” towards HIV sexual risk behavior [17–19], but this has yet to be measured among MWID. Additionally, migrant-related stressors including both loneliness and depression have been linked to HIV risk behavior [20–22]. Among Tajik MWID, poor working and living conditions, loneliness due to separation from family and friends at home, and Tajik gender constraints to condom use all reflect the general emotional stress common to temporary migrant laborers in diaspora and an increased vulnerability to HIV through engaging in risky sexual behavior [5, 6, 23]. Examining factors associated with sexual risk behaviors among male Tajik MWID as an understudied population may help to inform prevention strategies for successfully addressing their sexual risk while adding to a better scientific understanding of the relationship between these key factors and risky sexual behavior.

### Methods

The research design and procedures of this study were reviewed and approved by the institutional review boards of the University of Illinois Chicago, PRISMA Research Center, and the Moscow Nongovernment Organization, “Bridge to the Future.” The analysis is based on data collected at baseline for a clinical trial assessing the efficacy of the “Migrants’ Approached Self-Learning Intervention in HIV/AIDS for Tajiks” (MASLIHAT) peer-education model in reducing HIV risk behavior among Tajik MWID while living in Moscow.

### Recruitment

From October 2021 to April 2022, 140 male Tajik migrant workers who inject drugs were recruited from 12 sites in Moscow: 2 Tajik diaspora organizations, 4 bazaars, and 6 construction work sites. To be eligible to participate in the research, prospective participants had to be a male Tajik migrant aged 18 or older, a current or former PWID, give written informed consent, intend to reside in Moscow for the next 12 months to permit completing baseline and four follow-up interviews, and willing to recruit two male Tajik migrants who inject drugs for interviewing as PWID network members. Network members (*n* = 280) had to meet the same eligibility criteria as the migrant who referred them but also: 1) have injected drugs at least once in the last 30 days; and 2) be someone whom the referring migrant sees at least once a week to facilitate sharing MASLIHAT HIV prevention information. The analysis draws on data collected from the study’s sample of 420 male Tajik MWID in Moscow at enrollment (baseline) prior to their random assignment to one of two research intervention conditions.

### Data collection

After giving informed consent, participants were interviewed at the PRISMA office in Moscow or a private location of their choosing. A structured questionnaire collected information on participants’ sociodemographic characteristics, HIV-related knowledge, HIV risk perception, substance use and sexual risk behavior, experience with police-enacted stigma, and psychosocial measures of depression and loneliness. Participants received the customary compensation in Moscow of $20.00 for their time and transportation costs in being interviewed*.*

### Measures

Demographic information consisted of age (years), marital status (currently married, not married/divorced), and highest educational attainment (primary school/secondary school, some university education/higher).

HIV knowledge was assessed by responses to 8 statements as being either “safe” or “unsafe” in the possibility of HIV transmission such as “being bitten by mosquitoes or other insects” and 5 statements as either “true” or “false” in terms of becoming infected such as “there is a test to determine if a person has HIV.” Responses were coded as either correct or incorrect with “don’t know” coded as incorrect. Correctly answered items were summed for a possible final score per individual between 0 – 13 (Cronbach’s alpha: 0.91). Low HIV knowledge was defined as scoring from 0 – 6 and moderate/high as scoring from 7–13.

Self-perception of HIV risk was assessed with two items: 1) “How likely are you to get HIV?” on a scale from “not at all likely” (0) to “very likely” (3). 2) How much do you worry about HIV?” from “not at all” (0) to a lot (2). A self-perceived HIV risk score was created per person by summing the responses to both items with scores ranging from 0—5. Low HIV risk perception was defined as a score of 0 or 1 and moderate/high as scoring between 2–5.

HIV sexual risk behavior was measured with three items: number of female sex partners, sex with female sex workers (FSW), and engaging in condomless sex. To assess possible HIV risk behavior through sexual partnering, participants were asked how many women and the number of FSW with whom they had sexual intercourse in the past 30 days. Number of female sex partners was coded as: 0 or 1 sex partner = 0 (little to no risk) or ≥ 2 sex partners = 1 (some level of risk). Sex with FSW was coded as: 0 FSW = 0 (no sexual intercourse reported with FSW) or ≥ 1 FSW = 1 (Sex with FSW). To assess frequency of engaging in sex without a condom: participants were asked, “how often did you use a condom when having sexual intercourse?” for each of three partner categories: “regular female partner in Russia,” “Moscow FSW,” and “other sexual partners not engaged in selling sex.” Response categories were “never,” “sometimes,” “often,” or “always.” Responses of “always used a condom” for all three partner categories were categorized as “engaging in sex with condoms.” Otherwise, responses were categorized as engaging in condomless sex. The items were combined to create a binary measure of “any condomless sex” vs. “sex with condoms” in the past 30 days.

### Psychosocial measures

Symptoms of depression were measured using the 20-item Center for Epidemiologic Studies Depression scale—revised (CESD-R) (Cronbach’s alpha: 0.91) [24, 25]. Based on the total score as calculated by the sum of item responses and responses to select items, a depression score was created with five ordinal categories: 1) no clinical significance, 2) subthreshold depression symptoms, 3) possible major depressive episode, 4) probable major depressive episode, and 5) meets criteria for major depressive episode. Loneliness was measured using the 20-item UCLA loneliness scale and a loneliness score was calculated as the mean of item responses (Cronbach’s alpha: 0.94) [26].

Police-enacted stigma was measured by responses ranging from “never” (0) to “very often” (4) to each of three statements: “I have been hassled by the police because I’m a migrant,” “I have been detained by the police because I’m a migrant,” and “I have been beaten by the police because I’m a migrant.” A summation score of experience with police-enacted stigma was calculated per participant ranging from 0–12.

### Analysis

A population-averaged modified Poisson regression analysis was conducted with a sandwich estimator of variance and exchangeable within-group correlation structure for network clusters to obtain adjusted prevalence ratios (aPR) with their 95% confidence intervals (95% CI) [27]. Adjusting for demographic and psychosocial factors that might impact HIV sexual risk behavior, multivariable modeling was used to examine associations between HIV knowledge and HIV risk perception and four sexual risk outcomes: sex with multiple partners (model 1), sexual activity with one or more FSW (model 2), condomless sex (model 3) and condomless sex with FSW (Model 4). To test the possible moderating effect of HIV risk perception on the relationship between HIV knowledge and sexual risk behavior, the analysis tested for both the separate and interactive effects of HIV knowledge and perception of HIV risk on each sexual outcome. The analyses were performed using Stata software (v. 18) [28].

## Results

Table 1 presents both the demographic characteristics of the total sample and the subsample of participants reporting sexual activity in the 30 days prior to being interviewed. The total sample of 420 participants was on average 30 years of age, not married/divorced, and at a secondary school education level. No significant differences were found between the demographic characteristics of men recruited through worksites, bazaars, and non-governmental organizations (NGO) versus those whom they referred to the study as network members with the exception that the latter on average were one year younger (B = -1.07, 95% CI -1.69 – -0.46) and more likely to be on their first labor migration trip (Wald χ^2^ = 7.49, *p* = 0.02).

**Table 1:** Demographic Characteristics & HIV Sexual Risk Behavior of Male Tajik MWID

**Study Variable**^a^	**Total Population, *N* = 420**			**Among Those Reporting Sexual Activity, *N* = 255**		
	***Range***	***Mean***	***SD***	***Range***	***Mean***	***SD***
*Demographic Factors*						
Age	19–50	29.99	6.20	19–50	27.98	5.61
		***n***	***%***		***n***	***%***
Married	–	52	12.4	–	19	7.5
Highest Education						
Primary	–	16	3.8	–	7	2.8
Secondary	–	240	57.1	–	177	69.4
College or Technical	–	105	25.0	–	54	21.2
University (No degree)	–	14	3.3	–	6	2.4
University Degree	–	45	10.7	–	11	4.3
*Study Factors*						
HIV Knowledge Score	0–13	7.21	3.30	0–13	7.05	3.32
HIV Risk Perception Score	0–5	1.75	1.35	0–5	1.61	1.17
		***n***	***%***		***n***	***%***
Low HIV Knowledge	–	136	32.4	–	88	34.5
Low HIV Risk Perception	–	189	46.1	–	123	49.2
Loneliness	19–68	43.54	12.33	19–68	41.23	12.14
Depression	0–4	0.90	0.97	0–4	0.76	0.83
Police-Enacted Stigma	0–8	2.70	2.05	0–8	2.58	2.06
*Study Outcomes*						
		***n***	***%***		***n***	***%***
Multiple Sex Partners^b^	–	125	29.8	–	124	48.6
Sex with FSW	–	177	42.1	–	177	69.4
Condomless Sex	–	178	42.4	–	178	69.8
Condomless Sex with FSW	–	62	14.8	–	62^c^	35.0

^a^ Frequency of study characteristics may not sum to column N due to missingness

^b^ One individual reporting multiple sex partners did not complete information to assess condomless sex

^c^ Among those reporting sex with FSW, *N* = 177

### Sexual risk behavior

Of the total 420 participants in the study sample, 60.7% (255) reported having been sexually active in the last 30 days prior to being interviewed. Of these, 124 men (48.6%) reported having sex with multiple partners, 177 (69.4%) reported sex with one or more FSW, and 178 (69.8%) reported engaging in sex without a condom including 62 men (35%) who did not use one when having sex with FSW.

### HIV knowledge

Thirty-two percent of participants (*n* = 136) scored low on HIV knowledge. Of the 88 sexually active men who also scored low on HIV knowledge, 45 (51.1%) reported having multiple partners, 62 (70.5%) reported sex with FSW, and 61 (69.3%) reported engaging in condomless intercourse including 22 participants who reported not having used one with FSW. Comparing those with low to moderate/high HIV knowledge scores, sexual risk behavior was not significantly different. Of the 167 sexually active men who scored moderate/high on HIV knowledge, 79 (47.3%) reported having multiple partners, 115 (68.9%) reported sex with FSW, and 117 (70.1%) reported engaging in condomless sex including 40 men who reported sexual activity with FSW.

### Self-perception of HIV risk

Forty-five percent of participants (*n* = 189) perceived themselves to be at low risk for HIV. Of the 123 sexually active men who also perceived themselves to be at low HIV risk, 82 (66.7%) reported having multiple sex partners, 97 (78.9%) reported sexual activity with FSW, and 83 (67.5%) reported engaging in condomless sex including 39 men who reported sexual activity with FSW. Comparing those with low to moderate/high HIV risk perception scores, reporting multiple sex partners and reporting sexual activity with FSW were significantly different (*P* < 0.05), but reporting engaging in condomless sex including condomless sexual activity with an FSW were not significantly different. Of the 127 sexually active men who also perceived themselves to be at moderate/high HIV risk, 39 (30.7%) reported having multiple sex partners, 75 (59.1%) reported sexual activity with FSW, and 92 (72.4%) reported engaging in condomless sex including 21 men who reported sexual activity with FSW.

### *Multivariable* analyses

All study participants for whom there were complete data were included in the multivariable modeling of factors associated with engaging in sexual risk. Table 2 shows the adjusted prevalence ratios of each sexual risk behavior. Model 1 and 2 examine the association between key variables and engaging in sex with multiple sex partners and with FSW. Low HIV risk perception was associated with a 79% higher prevalence of multiple sex partners (aPR: 1.79, 95% CI: 1.34, 2.40, Table 2) and a 28% higher prevalence of sexual activity with FSW (aPR: 1.28, 95% CI: 1.04, 1.59) when compared to those with moderate/high HIV risk perception. Older age was associated with a lower prevalence of multiple sex partners (aPR: 0.87, 95% CI: 0.84, 0.91) and sexual activity with FSW (0.91, 95% CI: 0.88, 0.94). A one-unit increase in depression scoring was associated with a 19% lower prevalence of multiple sex partners (aPR: 0.81, 95% CI: 0.65, 1.01). Conversely, a one-unit increase in police-enacted stigma score was associated with a 22% greater prevalence of multiple sex partners (aPR: 1.22, 95% CI: 1.01, 1.49) and a 32% greater prevalence of sexual activity with FSW (aPR: 1.32, 95% CI: 1.13, 1.54).

**Table 2:** Adjusted Associations with Number of Sex Partners and Condomless Sex Among Male Tajik MWID

Study Variable	Model 1: Multiple Sex Partners, *N* = 410	Model 2: Sex with FSW, *N* = 410	Model 3: Condomless Sex, *N* = 250	Model 4: Condomless Sex with FSW, *N* = 172
	***aPR (95% CI)***	***aPR (95% CI)***	***aPR (95% CI)***	***aPR (95% CI)***
Low HIV Knowledge	0.92 (0.69, 1.21)	0.98 (0.79, 1.20)	1.03 (0.87, 1.21)	0.92 (0.63, 1.34)
Low HIV Risk Perception	1.79 (1.34, 2.40)^**^	1.28 (1.04, 1.59)^*^	0.99 (0.84, 1.16)	1.49 (0.88, 2.52)
Age	0.87 (0.84, 0.91)^**^	0.91 (0.88, 0.94)^**^	1.02 (1.01, 1.03)^**^	1.04 (0.99, 1.09)
Loneliness	1.06 (0.87, 1.31)	1.18 (0.96, 1.44)	0.79 (0.68, 0.92)^**^	0.82 (0.52, 1.31)
Depression	0.81 (0.65, 1.01)	0.88 (0.75, 1.02)	1.14 (1.05, 1.24)^**^	1.26 (1.03, 1.54)^*^
Police-Enacted Stigma	1.22 (1.01, 1.49)^*^	1.32 (1.13, 1.54)^**^	0.91 (0.80, 1.04)	1.28 (0.92, 1.79)
Married	0.38 (0.14, 0.99)^*^	0.70 (0.45, 1.08)	0.74 (0.48, 1.15)	0.33 (0.04, 2.55)
Highest Education				
Primary/Secondary	1.00	1.00	1.00	1.00
Some College/Above	0.78 (0.58, 1.04)	0.87 (0.68, 1.11)	0.84 (0.67, 1.05)	0.79 (0.48, 1.28)

^*^Denotes *p* < 0.05

^**^Denotes *p* < 0.01

Model 3 and Model 4 investigate condomless sex generally as well as specifically with FSW. Neither knowledge of how HIV is transmitted, nor HIV risk perception were significantly associated with condomless sex or condomless sex with FSW. Meanwhile, higher levels of loneliness were associated with less likelihood of engaging in condomless sex (aPR: 0.79, 95% CI: 0.68, 0.92), while a one-unit increase in depression score was associated with a 14% higher likelihood of engaging in condomless sex (aPR: 1.14, 95% CI: 1.05, 1.24) and a 26% higher likelihood of engaging in condomless sex with FSW specifically (aPR: 1.26, 95% CI: 1.03, 1.54).

The joint effect of HIV knowledge and HIV risk perception on each sexual risk outcome is shown in Table 3. No evidence of an additive interaction between HIV knowledge and HIV risk was found. When low HIV risk perception was present, however, the prevalence of multiple sex partners, sexual activity with FSW, and condomless sex with FSW was higher. However, significant moderating effects were only found for multiple sex partners.

**Table 3:** Joint Effect of HIV Knowledge and HIV Risk Perception Among Male Tajik MWID

**HIV Knowledge**	**HIV Risk Perception**	**N**	**% Reporting Outcome**	**Prevalence Ratio aPR (95% CI)**^a^	**Relative Excess Risk due to Interaction (95% CI)**
*Model 1: Multiple Sex Partners, ****N* = 410**					
Moderate/High	Moderate/High	26	16.4	ref	–
Low	Moderate/High	14	22.6	0.94 (0.34, 1.45)	–
Moderate/High	Low	52	44.1	1.82 (1.21, 2.76)^*^	–
Low	Low	30	42.3	1.65 (1.01, 2.74)^*^	-0.11 (-0.70, 0.60)
*Model 2: Sex with FSW, ****N* = 410**					
Moderate/High	Moderate/High	52	32.7	ref	–
Low	Moderate/High	23	37.1	0.89 (0.55, 1.28)	–
Moderate/High	Low	59	50.0	1.19 (0.87, 1.49)	–
Low	Low	38	53.5	1.21 (0.89, 1.70)	0.13 (-0.27, 0.64)
*Model 3: Condomless Sex, ****N* = 250**					
Moderate/High	Moderate/High	61	70.9	ref	–
Low	Moderate/High	31	75.6	1.06 (0.88, 1.26)	–
Moderate/High	Low	53	68.8	1.01 (0.90, 1.30)	–
Low	Low	30	65.2	0.96 (0.72, 1.24)	-0.10 (-0.58, 0.15)
*Model 4: Condomless Sex with FSW, ****N* = 172**					
Moderate/High	Moderate/High	14	26.9	ref	–
Low	Moderate/High	7	30.4	1.14 (0.40, 2.29)	–
Moderate/High	Low	24	40.7	1.68 (0.80, 3.61)	–
Low	Low	15	39.5	1.63 (0.79, 2.93)	-0.19 (-2.22, 1.05)

^a^All estimates adjusted for study variables in modified Poisson regression

^*^ Denotes *p* < 0.05

## Discussion

To the best of our knowledge, this is the largest cross-sectional study to examine the factors that may influence HIV sexual risk behavior among male Tajik MWID, a highly understudied population at-risk for HIV [29]. Prevalence of HIV sexual risk behavior among this migrant population is high as evidenced by the number of men engaging in sex with multiple partners (48.6%) and sex without a condom (69.8%) including with FSW (35.0%). Of the total study sample, 32.4% scored low on HIV knowledge and 46.1% on self-perceived HIV risk. The prevalence of HIV sexual risk behavior in this study sample is consistent with a cross-sectional study among male Tajik migrants in Moscow [30], but higher than a second study that included both Eastern European and Central Asian (EECA) male labor migrants in Moscow [31]. In this cross-sectional study, thirty percent of migrants reported multiple female partners and condom use ranged from 35 to 52% [31]. Possibly due to male Tajik gender norms and/or other challenges unique to the Tajik migrant experience in Moscow [23], male Tajik migrants, especially male Tajik MWID, may have greater sexual risk for HIV acquisition than their EECA counterparts [3].

Prevention programs for at-risk populations typically build on the assumption that knowledge of which sexual activities carry HIV risk is needed to reduce or end high risk behavior [32, 33]. Yet, the study’s finding that HIV knowledge was not associated with engaging in risky sexual behavior suggests that providing male Tajik migrants with HIV risk-reduction education alone likely would prove ineffective. Meanwhile, low self-perceived risk for HIV was found to be associated with greater likelihood of engaging in risky sexual activity as indicated by multiple partners and condomless sex including with FSW. Analysis of the possible joint influence of HIV knowledge and self-perceived risk for HIV on risky sexual behavior failed to support an interaction effect. These findings suggest the role of self-perceived HIV risk over HIV knowledge as a significant factor influencing sexual risk behavior. These findings are critical as the scope of prevention work in Moscow is often restricted to bolstering individual knowledge and behaviors through peer education interventions. This is due to the lack of access of preventive services, such as pre-exposure prophylaxis and methadone, and citizen requirements needed for accessing HIV care in Russia [10, 34], HIV prevention programming designed to raise Tajik migrants’ and possibly other vulnerable populations’ awareness of being at personal risk is particularly needed to strengthen the effects of HIV preventive interventions.

As for psychosocial-related factors, police-enacted stigma was associated with sexual activity with multiple sex partners and FSW in the last 30 days whereas depression was associated with condomless sex both in general and specifically with FSW. These findings add to the documented role of both police-enacted stigma and depression to be associated with HIV sexual risk behavior among Tajik labor migrants [35–38]. To contextualize intersectional stigma, effective HIV prevention programming among Tajik labor migrants should specifically aim to alleviate the pressure of police aggression and harassment on overall wellbeing, consistent with a separate study that recognizes the role of policing in HIV prevention programming among labor migrants [17]. In addition to police-enacted stigma, depression may largely be explained by the overall challenges of migrant life in Russia [8, 23, 31]. However, loneliness was associated with a lower prevalence of condomless sex among sexually active male MWID, contrary to the documented role of loneliness as a sexual risk factor among migrant populations [20, 38]. Possibly among male migrants who are not sexually active and/or experience loneliness, these negative feelings can manifest themselves in either the decreased likelihood of engaging in any sexual activity or more protective attitudes towards sex. The correlation of these psychosocial factors with risky sex calls attention to the need for HIV prevention programming that helps to address the stress and psychosocial challenges of migrant life. Additionally, the prevalence of all psychosocial variables as reported in Table 1 was marginally higher among men who disclosed not having engaged in sex versus those who were sexually active during the same 30-day period. The similarity between the two sub-populations suggests that Tajik migrants in general are subject to emotional distress that can negatively affect their daily lives.

### Limitations

The study’s analysis of the prevalence and correlates of risky sex focuses solely on those who reported sexual activity and excludes those men who reported being sexually inactive. It may be that some unknown number of the study’s sexually inactive participants purposely abstained from sex to avoid HIV, but the study’s data do not include inquiry into this possibility. Also, neither of the two measures used in asking participants to assess their personal risk for HIV specified that this calculation should be based solely on sexual risk. Given that the study’s sample is composed entirely of PWID, some proportion of participants may have factored in personal risk for HIV through both sex and injection drug use. While this likelihood doesn’t mitigate the validity of the study’s findings derived by examining the association between self-perceived risk for HIV and engaging in risky sexual behavior, it does not allow assessment of how much drug use alone may contribute to Tajik MWID’s self-perception of being at HIV risk.

In terms of sampling, the study’s initial participants were obtained through in-person recruitment at 10 occupational sites and by referral from 2 Tajik service networks. Each of these participants, in turn, recruited two active MWID participants through their social networks. Consequently, the results of the study may not generalize to those migrants whom these methods failed to reach or who chose not to participate. Furthermore, the study’s measures of sexual risk behavior focus solely on unsafe sex with female sexual partners. It is quite possible that acts of unsafe sex with another male also may have occurred during the same 30-day period. Same-sex behavior is highly stigmatized and considered morally unacceptable within the male Tajik migrant community, and it is unlikely that its occurrence would be disclosed by a participant during an initial interview. Asking a male Tajik about possibly having engaged in same-sex behavior is sufficiently affrontive culturally that it carries the potential risk of triggering the participant’s decision to end the interview. Consequently, measures of sexual risk in the study are confined solely to unsafe sex with female partners.

For the multivariable analysis, differences in findings between models may be partly explained by the differences in sample sizes; the sample of those who report sexual activity, and specifically sexual activity with FSW, may differ from the overall sample. Model 3 and Model 4 are subject to a lower precision due to a smaller sample compared to Model 1. This is evident in the assessment for the moderating effect of HIV risk perception as both Model 1 and Model 4 noted a similar increase in magnitude for the respective outcomes (multiple sex partners and sexual activity with FSW) when HIV risk perception was low, but only Model 1 was significant. Finally, the study’s baseline data are cross-sectional and only examined HIV risk behavior in the last 30 days. They cannot provide information about how variables of interest may have differed prior to or after this measurement period.

## Conclusions

The study’s results underline the need for prevention programing for male Tajik MWID who have high levels of sexual risk behavior that go beyond increasing HIV knowledge to also promote evidence-based awareness of personal risk for HIV. Additionally, psychological services to counter the psychosocial effects of depression, and police-enacted stigma are needed.

## Data Availability

The data that support the findings of this study are openly available at 10.17605/OSF.IO/WS5MP [39].

## Abbreviations

HIV: Human immunodeficiency virus
MWID: Migrants who inject drugs
PWID: People who inject drugs
AIDS: Acquired immunodeficiency syndrome
MASLIHAT: Migrants’ Approached Self-Learning Intervention in HIV/AIDS for Tajiks
FSW: Female sex workers
CESD-R: Center for Epidemiologic Studies Depression scale – revised
aPR: Adjusted prevalence ratios
95% CI: 95% Confidence intervals
NGO: Non-governmental organizations
EECA: Eastern European and Central Asian
